# High expression of *gabarapl1* is associated with a better outcome for patients with lymph node-positive breast cancer

**DOI:** 10.1038/sj.bjc.6605568

**Published:** 2010-03-02

**Authors:** A Berthier, S Seguin, A J Sasco, J Y Bobin, G De Laroche, J Datchary, S Saez, C Rodriguez-Lafrasse, F Tolle, A Fraichard, M Boyer-Guittaut, M Jouvenot, R Delage-Mourroux, F Descotes

**Affiliations:** 1 Université de Franche-Comté, UFR Sciences et Techniques, IFR 133, Besançon EA3922, France; 2 Epidemiology for Cancer prevention, Inserm U 897 Université Victor Segalen Bordeaux 2, Bordeaux, France; 3 Centre Hospitalier Lyon Sud, Hospices Civils de Lyon, Pierre Bénite, France; 4 Clinique Mutualiste, St Etienne, France; 5 Centre Hospitalier Régional, Annecy, France

**Keywords:** breast cancer, *gabarapl1*, prognosis, lymph node positive

## Abstract

**Background:**

This study evaluates the relation of the early oestrogen-regulated gene *gabarapl1* to cellular growth and its prognostic significance in breast adenocarcinoma.

**Methods:**

First, the relation between *GABARAPL1* expression and MCF-7 growth rate was analysed. Thereafter, by performing macroarray and reverse transcriptase quantitative-polymerase chain reaction (RT–qPCR) experiments, *gabarapl1* expression was quantified in several histological breast tumour types and in a retrospective cohort of 265 breast cancers.

**Results:**

GABARAPL1 overexpression inhibited MCF-7 growth rate and *gabarapl1* expression was downregulated in breast tumours. *Gabarapl1* mRNA levels were found to be significantly lower in tumours presenting a high histological grade, with a lymph node-positive (pN+) and oestrogen and/or progesterone receptor-negative status. In univariate analysis, high *gabarapl1* levels were associated with a lower risk of metastasis in all patients (hazard ratio (HR) 4.96), as well as in pN+ patients (HR 14.96). In multivariate analysis, *gabarapl1* expression remained significant in all patients (HR 3.63), as well as in pN+ patients (HR 5.65). In univariate or multivariate analysis, *gabarapl1* expression did not disclose any difference in metastasis risk in lymph node-negative patients.

**Conclusions:**

Our data show for the first time that the level of *gabarapl1* mRNA expression in breast tumours is a good indicator of the risk of recurrence, specifically in pN+ patients.

Breast cancer is the most frequently diagnosed cancer among women worldwide, with more than 1.3 million cases each year. The understanding of this disease has progressed considerably and its prognosis has improved because of earlier diagnosis, the introduction of appropriate strategies and the use of novel active treatments (Aapro, 2001; Sasco *et al*, 2003). However, as the tumour-node–metastasis (TNM) stage provides scant information on the growth pattern of each tumour, a large number of new biomarkers have been analysed to predict the risk of recurrence and to help apply the best adjuvant therapy. In this view, we paid attention to a recently identified oestrogen-regulated gene called *gabarapl1* (*GABA*_*A*_ *receptor-associated protein-like 1*) or *gec1* (*glandular epithelial cell 1*), which is thought to have an essential role during tumour progression (Nemos *et al*, 2003).

The *gabarapl1* gene was originally identified as an early oestrogen-regulated gene in cultured guinea-pig endometrial glandular epithelial cells (GECs) (Pellerin *et al*, 1993). The human gene was then characterised (GeneBank Accession No. AF087847) and its coding sequence presents 76.8% identity with that of *gabarap* (*γ-aminobutyric acid type A receptor-associated protein*). Indeed, *gabarapl1* and *gabarap* genes are located on 12p12.3 and 17p13.12 human chromosomes, respectively.

The GABARAPL1 protein is composed of 117 amino acids and is highly conserved throughout evolution, suggesting a critical cellular function. Similar to GABARAP, GABARAPL1 is involved in protein or vesicle intracellular transport through its interaction with cytoskeleton elements. Some publications have suggested that GABARAPL1 and GABARAP might also be involved in tumour development. Indeed, it was reported that lower levels of *gabarap* gene expression predict decreased survival among patients with neuroblastoma (Roberts *et al*, 2004). Klebig *et al* (2005) showed that an ectopic overexpression of the *gabarap* gene inhibits cancer cell proliferation and tumour growth in mice. We reported elsewhere a decrease in *gabarapl1* expression in cancer cell lines (Nemos *et al*, 2003).

To characterise the role of *gabarapl1* in breast cancer, we analysed the level of *gabarapl1* expression in some breast tumour samples and the effect of its induced overexpression on the growth rate of a breast cancer cell line. We also analysed *gabarapl1* mRNA expression in a retrospective cohort of 265 breast tumour biopsy samples using a reverse transcriptase–quantitative polymerase chain reaction (RT–qPCR) protocol to estimate its potential prognostic effect.

## Materials and methods

### Experimental analysis

#### Cell transfection

Human breast cancer cells (MCF-7) were maintained as previously described (Berthier *et al*, 2007). The *gabarapl1* coding sequence flanked by two tag sequences coding for a Flag peptide and a six-histidine tail was cloned into a pcDNA3.1 Hygro(−) vector (Invitrogen, Carlsbad, CA, USA). This construct was called pcDNA3.1-Flag-GEC1-(His)_6_. MCF-7 cells were transfected with 40 *μ*g of pcDNA3.1-Flag-GEC1-(His)_6_ or pcDNA3.1 control vector, and 40 *μ*l of TransFast reagent (Promega, Madison, WI, USA) according to the manufacturer's protocol. The selection of resistant cells was carried out for 20 days with 200 *μ*g/ml hygromycin starting 24 h after transfection until single colonies could be picked.

#### Western blot analysis

Whole-cell lysates (40 *μ*g) were loaded on a 12% SDS–PAGE and a western blot analysis was performed according to the standard protocol (Towbin *et al*, 1979). A monoclonal anti-Flag antibody was used at 1/6000 dilution (Sigma-Aldrich, St Louis, MO, USA). Signals were visualised using a goat horseradish peroxidase (HRP)-coupled anti-mouse antibody (1/20 000) (PARIS, Compiègne, France) and the ECLplus reagent (GE Healthcare Life Sciences, Pittsburgh, PA, USA) according to the manufacturer's protocol. Three independent experiments were performed for each cell lysate.

#### Cell proliferation assay

MCF-7-Flag-GEC1-(His)_6_ (clones 1 and 2) and MCF-7-pcDNA cell lines were plated in 96-well plates (3000 cells per well) and cell proliferation experiments were conducted over a 10-day period using 3-(4,5-dimethylthiazol-2-yl)-2,5-diphenyl tetrazolium bromide (MTT) (Sigma-Aldrich) (Morel *et al*, 2007). For each clone, two independent experiments were performed in 16 wells. Data are means±s.d. and differences between clones were assessed using the Wilcoxon test (R software version 2.7.1, http://cran.r-project.org).

#### Macroarray experiment

The macroarray experiment was performed using a cancer profiling array II membrane (Clontech, Palo Alto, CA, USA). A 319-bp *gabarapl1* probe, specific for the 3′ mRNA untranslated region, was prepared as previously described (Nemos *et al*, 2003). A volume of 50 ng of *gabarapl1* probe was denaturated (10 min at 95 °C) and randomly labelled (1 h at 25 °C) with 50 *μ*Ci of *α*[^32^P]-dCTP (Random Primer DNA Labelling System; Invitrogen). The membrane was incubated overnight with the *gabarapl1* probe according to the manufacturer's protocol, exposed for 30 h in a Storm 840 PhosphorImager (Molecular Dynamics, Sunnyvale, CA, USA) and signals were quantified using ImageQuant TL v2005 software (GE Healthcare Life Sciences). For macroarray normalisation, the membrane was stripped according to the manufacturer's protocol and hybridised with a control ^32^P-labelled *ubiquitin* probe.

### Clinical analysis

#### Patients and tumour characteristics

Patients treated in three medical centres (Centre Hospitalier Régional Annecy, Chirurgie Oncologique Centre Hospitalier Universitaire Lyon-Sud and Clinique Mutualiste Saint Etienne, France) were included between October 1994 and October 2001 (*n*=265; Table 1). Patients were selected according to the following criteria: primary breast tumour without inflammatory features, no previous treatment and no evidence of distant metastasis at the time of diagnosis (Descotes *et al*, 2008). The median age at primary surgery was 53 years (range 29–89). The tumour type was determined according to the UICC-WHO criteria (Sobin and Wittekind, 1997) and histological grading was scored according to the Scarff Bloom and Richardson classification (Bloom and Richardson, 1957) only in the ductal carcinomas that represented the majority (81.5%) of cases. Oestrogen receptor (ER) and progesterone receptor (PgR) were assayed in cytosol using the radioligand reference method (EORTC, 1980). Quality control was based on regular testing of both European Organization for Research and Treatment of Cancer (EORTC) and internal controls. Results were expressed as fmol per mg cytosol protein. ER- and PgR-positive tumours contained >2 and >5 fmol per mg protein, respectively. All patients received locoregional radiotherapy. The majority of node-positive patients and high-risk node-negative patients (age of <35 years, pathological size >20 mm, histological grade of ⩾2 and steroid receptor-negative status) received chemotherapy. Almost all ER-positive patients were given hormone treatment.

#### RT–qPCR analysis

Breast cancer tissue biopsy samples were obtained by surgery, selected by the pathologist and immediately stored in liquid nitrogen until processing. The biopsy samples were pulverised using a ‘Mikro-Dismembrator’ (B. Braun Biotech International, Melsungen, Germany) and total RNAs were extracted using TRI Reagent (Sigma). To remove any genomic DNA contamination, total RNAs were treated with RNAse-free DNAse I and purified using RNeasy microcolumns (Qiagen, Hilden, Germany). RNA quality was verified using an Agilent Bioanalyser 2100 (Agilent Technologies, Santa Clara, CA, USA). A volume of 500 ng of total RNAs was reverse transcribed using M-MLV RT RNase H Minus reverse transcriptase and oligo(dT)_15_ primer following the manufacturer's instructions (Promega). All cDNA amplifications were performed using 1/20th of the reverse transcription products and the LC Fast Start DNA Master SYBR Green kit (Roche Applied Science, Basel, Switzerland), in the presence of 3 mM MgCl_2_ and 0.4 *μ*M of each *gabarapl1* primer. Quantitative PCR was run on a LightCycler instrument (Roche Applied Science) with the following parameters: 10 min at 95 °C for the initial denaturation step, followed by 15 s at 95 °C, 6 s at 60 °C and 12 s at 72 °C per cycle for a total of 40 cycles. The *gabarapl1* primers used (forward: 5′-TTTGGTGCCCCTTATCTCAC-3′ reverse: 5′-GGCCATCATGTAGCATTCCTT-3′) for amplification of a 241-bp fragment (GenBank AF287012) were designed using the Primer3 software (http://fokker.wi.mit.edu/primer3/input.htm). The amplified cDNA concentration was evaluated using an external curve of standard samples and specific amplification was checked using a melting curve. The PCR kinetics and quantitative data were determined using LightCycler software 4.05 (Roche Applied Science). The *gabarapl1* target concentration was expressed relative to the concentration of the *gapdh* housekeeping gene. The forward primer (5′-CGACCACTTTGTCAAGCTCA-3′) and the reverse primer (5′-AGGGGAGATTCAGTGTGGTG-3′) gave an amplification product of 203 bp (GenBank NM_002046). Quality control was assessed using regular testing of two internal controls. Interassay variations were <5% (data not shown).

#### Statistical analysis

The median follow-up at the time of analysis was 54 months (range 2–109). The criterion for statistical analyses was metastasis-free survival (MFS), that is, the delay between the time of primary surgery and the first event: nodal or distant metastasis, or death. Neither local recurrence nor occurrence of a contralateral cancer was taken into account, nor a second primary cancer if it occurred within 2 years. Patients alive without metastasis were censored at the last follow-up date. Analysis of the distribution of *gabarapl1* expression in relation to usual prognostic parameters was performed using the Mann–Whitney or Kruskall–Wallis test. Survival probabilities were estimated using Kaplan–Meier estimates and were compared using the log-rank test. Univariate and multivariate analyses were performed using the Cox proportional hazard model. When *gabarapl1* was used as a continuous variable, we used the transformed variable log(1/*gabarapl1*), which therefore provided an easier interpretation of the hazard ratio (HR). Multivariate analyses were performed in a stepwise forward manner. A basal model including the clinical, pathological and biological variables (except *gabarapl1*) associated with prognosis was first built. The histological grade that was determined only in ductal carcinomas but not in lobular carcinomas could not be introduced in the basal model. The variables were adjusted for age. The prognostic value of *gabarapl1* was tested after adding this variable to the basal model and the significance of each variable was calculated by comparing nested models using the likelihood ratio (LR) test. Trend tests were performed for ordinal variables. All tests were set at the significance level of *α*=0.05. Confidence intervals (CI) referred to the 95% level. These analyses were performed with the R software (release 2.7.1).

## Results

### Effect of GABARAPL1 overexpression on MCF-7 growth rate

**Figure 1:**
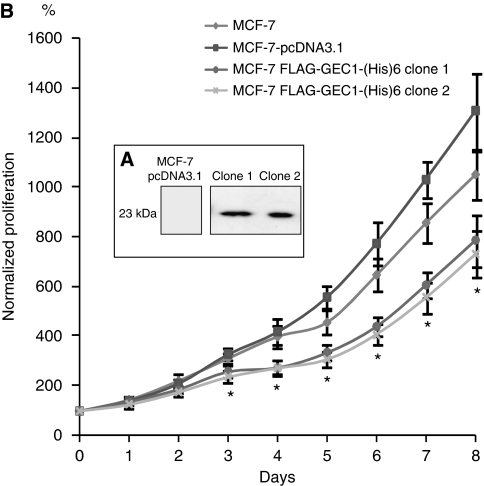
GABARAPL1 overexpression negatively regulates the growth rate of MCF-7 cells. (**A**) Western blot analysis of MCF-7-pcDNA3.1 (control) and MCF-7-Flag-GEC1-(His)_6_ (clones 1 and 2) cells using an anti-Flag antibody. (**B**) Growth rate of MCF-7 (▴), MCF-7-pcDNA3.1 (▪) and MCF-7-Flag-GEC1-(His)_6_ clones 1 (o) and 2 (
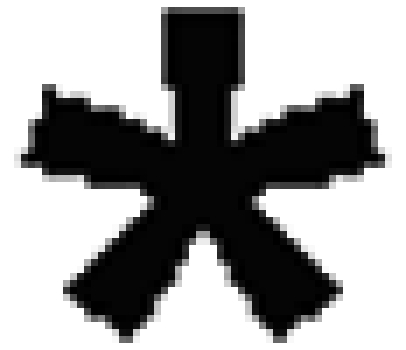
) using an MTT assay. ^*^P<0.05 (Wilcoxon's test).

Previous data have shown that *gabarapl1* mRNA is ubiquitously expressed in human tissues (Nemos *et al*, 2003), but surprisingly low levels were detected in some cancer cell lines, particularly in the MCF-7 breast cancer cell line (data not shown). Therefore, we speculated whether the ectopic expression of GABARAPL1 might modify the growth rate of these cells. To find a solution, we designed a stable MCF-7 cell line overexpressing the double-tagged Flag-GEC1-(His)_6_ protein. Among the hygromycin-resistant colonies, 20 clones were selected and GABARAPL1 protein expression was quantified by western blotting. As shown in Figure 1A, high GABARAPL1 levels were observed in clones 1 and 2 when compared with the control cell line. To assess whether overexpression of GABARAPL1 regulated the growth rate of breast cancer cells, we performed a kinetic viability assay (MTT) using wild-type MCF-7, MCF-7-pcDNA3.1 and two MCF-7-Flag-GEC1-(His)_6_-expressing clones. As shown in Figure 1B, GABARAPL1-expressing clones 1 and 2 showed significantly reduced growth rates over 8 days of culture when compared with control cell lines.

### Gabarapl1 expression in normal and tumour breast tissues

**Figure 2:**
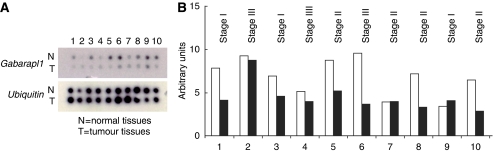
Macroarray hybridisation and analysis. Autoradiography of the membrane hybridised with (**A**) *gabarapl1* or *ubiquitin* probe. (**B**) Normalised *gabarapl1* expression using the *ubiquitin* signal in normal (white bars) and tumoural breast tissues (black bars). The tumours at different stages correspond to infiltrating ductal carcinomas (1, 2, 4, 7, 8, 9, 10), mucinous adenocarcinoma (3), Paget's disease (5) and lobular carcinoma (6).

As GABARAPL1 overexpression was associated with a decreased cancer cell growth rate, it can be expected that its expression might also be altered in tumour tissues. To test this hypothesis, we analysed *gabarapl1* expression in paired normal and tumour tissues using a cancer profiling array (Figure 2A). A dysregulation of *gabarapl1* expression was found not only in breast tumours but also in several other types of tumours such as kidney, testis, bladder, pancreas and prostate (data not shown). After normalisation with ubiquitin signal, these alterations in *gabarapl1* expression in tumour breast tissues were confirmed: a downregulation was detected in 7 out of 10 breast tumours (Figure 2B).

### Gabarapl1 expression in 265 breast cancer cases

To evaluate the significance of the macroarray data obtained on 10 breast tumours, we analysed *gabarapl1* expression in a cohort of 265 breast tumour biopsy samples. The mean *gabarapl1* value measured by RT–qPCR was 5.03 and the median was 4.54 (range 0.16–17.27). Table 2 shows the median value of g*abarapl1* in relation to several tumour characteristics that are usually linked to prognosis. Indeed, in the whole population, the median *gabarapl1* expression was significantly different in relation to surgical size, histological grade, lymph node, ER and PgR status. The histological type, ductal or lobular, revealed no difference. A lower *gabarapl1* expression was significantly related to tumour size of >20 mm only in the whole population and in the pN+ subset.

It may be observed that the median values were significantly lower in the pejorative categories of tumours. Therefore, after testing that the *gabarapl1* distribution was log normal (data not shown), for studies requiring a dichotomy of the variable, the cutoff value (6.56) was found to be equal to the upper threshold of the third quartile, allowing a discrimination between high and low *gabarap1l* expression status.

### Univariate analysis

Results of the univariate MFS analysis (Table 3) show the relation between *gabarapl1* expression levels and common prognostic factors: low levels were associated with pejorative prognostic factors. As usually observed, age and surgical tumour size were significant prognostic factors in the whole population and in the pN+ subset, but not in pN0 patients. In the whole population, lymph-node status was correlated with risk of metastasis (HR 3.67, *P*<0.001). It was also observed that the risk of metastasis in relation to low *gabarapl1* levels increased by 4.96-fold in the whole population (CI 2.43–10.12; *P*<0.001) and by 14.96-fold in the pN+ subset (CI 4.80–46.60; *P*<0.001). In pN0 patients, *gabarapl1* expression was not related to risk of metastasis.

**Figure 3:**
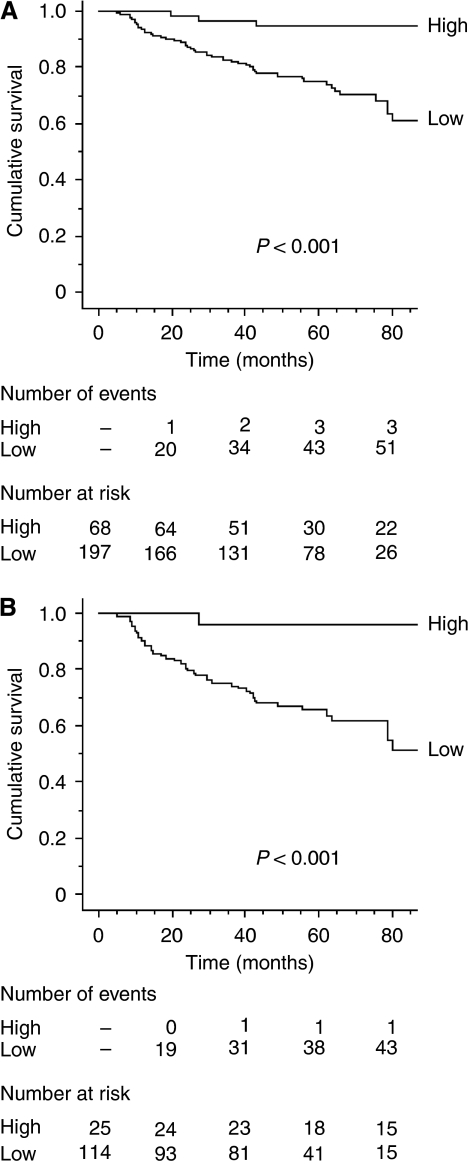
Kaplan–Meier curves for metastasis-free survival probabilities according to *gabarapl1* expression categorised with quartiles from (**A**) the whole population (*n*=265) and (**B**) pN+ patients (*n*=139). Low: ⩽75% quartile; high: >75% quartile.

Kaplan–Meier curves were constructed after segmentation into two groups on the basis of the *gabarapl1* expression cutoff (Figure 3). It was observed that high values of *gabarapl1* expression were related to a good prognosis. They were predictive of longer MFS in all patients (Figure 3A, *P*<0.001) and in pN+ patients (Figure 3B, *P*<0.001) but not in pN0 patients (data not shown). It is noteworthy that, in the high *gabarapl1* pN+ subgroup, only one patient relapsed.

### Multivariate analysis

In the whole population, the multivariate analysis applied to the basal model (Table 4) showed, as expected, a significantly higher risk of metastasis associated with surgical tumour size of >20 mm (HR 3.00; *P*=0.002), lymph node-positive status (HR 2.93; *P*=0.002) and ER and/or PgR-negative status (HR 2.15; *P*=0.007).

In pN+ patients, surgical tumour size of >20 mm (HR 3.59; *P*=0.004) and ER- and/or PgR-negative status (HR 2.92; *P*=0.001) were significantly related to higher metastasis risk, whereas none of these factors were related to the risk of metastasis in pN0 patients. When *gabarapl1* expression was included in this basal model, low *gabarapl1* values were associated with an increased metastasis risk by 3.63-fold in the whole population (CI 1.48–8.93, *P*=0.005) and by 5.65-fold in the pN+ subset (CI 1.84–17.29, *P*=0.002). It is observed that in pN0 patients, the risk of metastasis was not significantly related to *gabarapl1* expression levels.

## Discussion

In this study, we provide for the first time an insight into the effect of GABARAPL1 overexpression in breast cancer cells and into the effect of *gabarapl1* expression level in a large retrospective cohort of breast tumours.

We have reported that GABARAPL1 is able to bind to tubulin and could be involved in the transport of the GABA_A_ receptor (Mansuy *et al*, 2004). It also has an important role in the transport of other receptors such as the *κ*-opioïd receptor (Chen *et al*, 2006). Nevertheless, *gabarapl1* mRNA is widely distributed in human tissues (Nemos *et al*, 2003), suggesting that GABARAPL1 protein is not only involved in the transport of receptors but probably has a more complex role in cells. Particularly, it could be involved in cell cycle regulation, as it interacts with tubulin (Mansuy *et al*, 2004). Our study showed that MCF-7 cells overexpressing GABARAPL1 protein had a significantly decreased growth rate, compared with control cell lines. This finding was in favour of a potential role of GABARAPL1 in the control of cell proliferation as previously reported for GABARAP overexpression in CAL51 cells (Klebig *et al*, 2005). These data suggested that *gabarapl1* expression could be altered in rapidly growing breast tumour tissues. In fact, in the macroarray experiment, most breast tumour tissues presented weaker *gabarapl* mRNA levels than did normal tissues. These results concorded with previous findings in breast cancer cell lines, in which very low levels of *gabarapl1* mRNAs were observed (data not shown). Experiments are needed to determine whether somatic mutations (Tomlinson, 2001) or epigenetic events (Lo and Sukumar, 2008) are responsible for the *gabarapl1* downregulation in these cell lines.

*Gabarapl1* expression has been analysed in this study for the first time in a retrospective cohort of 265 breast tumours. The data obtained during this investigation showed that *gabarapl1* expression is significantly different in relation to usual prognostic criteria such as tumour size, lymph-node and steroid receptor status. It is also shown that *gabarapl1* expression median values are different in relation to tumour oestrogen and progesterone receptor status: the median is higher in ER- and PgR-positive tumours, in agreement with Mansuy *et al* (2004) who showed that *gabarapl1* is an oestrogen-regulated gene.

Furthermore, we showed that tumours expressing low levels of *gabarapl1* were observed to be significantly associated with high risk of metastasis in the pN+ subset (HR=14.96, *P*<0.001) but not in pN0 patients (Table 3). In the whole population, Kaplan–Meier curves (Figure 3A) showed that after 80 months of follow-up, only 3 out of 25 patients showing high *gabarapl1* levels presented a recurrence compared with 51 out of 77 patients with low *gabarapl1* levels. In the pN+ subset (Figure 3B), 1 out of 16 patients with a high *gabarapl1* level relapsed, compared with 43 out of 58 with a low *gabarapl1* level. These data clearly show that the *gabarapl1* expression level is negatively correlated with the risk of metastasis.

In this study, the difference in *gabarapl1* expression between ductal and lobular types, which show different growth patterns, is not significant. It can be observed that the median *gabarapl1* level is higher in lobular tumours, which are known to be less aggressive than ductal ones (Table 2). The difference in *gabarapl1* expression between both types is not significant, but it can be observed that the number of lobular tumours is very small. Moreover, we found a significant correlation between *gabarapl1* expression and the other pathological features related to prognosis, such as tumour size, histological grade, lymph node and ER and PgR status.

Previous results using RT–qPCR analysis on 235 neuroblastomas showed that lower GABARAP expression levels were associated with more advanced stages (Roberts *et al*, 2004). Moreover, tissue microarray experiments revealed a significant reduction in GABARAP protein expression in a high proportion of 93 breast cancers cases (Klebig *et al*, 2005). However, no correlation was observed between loss of GABARAP expression and clinicopathological features such as grading, tumour size, oestrogen receptor status and age of diagnosis. In the latter publication, the researchers used a polyclonal anti-GABARAP antibody (Alpha Diagnostics, San Antonio, TX, USA) to perform immunostaining of tissue microarrays. However, we have reason to believe that no commercially available antibody is able to clearly distinguish between GABARAP and GABARAPL1 proteins because of their high degree of identity. Indeed, all the polyclonal commercial and homemade antibodies we have tested so far in the laboratory recognised both GABARAPL1 and GABARAP proteins (Mansuy *et al*, 2004; Tolle *et al*, 2008). Therefore, immunostaining analysis is unreliable and, up to now, the unique alternative to differentiate *gabarapl1* and *gabarap* expression is the use of specific RT–qPCR primers located in 3′-untranslated regions.

It can be considered that despite their high homology, *gabarapl1* and *gabarap* are probably differently regulated during the course of breast cancer progression. Nevertheless, it would be of great interest to further study the expression levels of these two closely related genes to determine whether these present the same pattern of expression in breast cancers.

## Conclusions

Our data strongly suggest that, in breast cancers, high levels of *gabarapl1* mRNA are correlated with a low risk of metastasis. This is valid in the whole population, but specifically in lymph node-positive patients (HR 5.65; *P*=0.002). The *gabarapl1* gene might show an important effect on tumour progression. To our knowledge, all publications currently available only describe the role of GABARAPL1 protein during the intracellular transport of receptors in the brain. Hence, this investigation is the first one describing a new interesting function of this gene in breast tissues. These data open up a new point of view on the importance of this small protein called GABARAPL1 in different pathways and tissues and offer a great potential for this gene as a novel prognostic indicator for patients developing breast cancer.

## Figures and Tables

**Table 1:** Characteristics of the studied population

	**All patients**		**pN0 patients**		**pN+ patients**	
	***N*=265**		***N*=126**		***N*=139**	
**Characteristics**	***n***	**%**	***n***	**%**	***n***	**%**
*Age (years)*						
<47	71	26.8	30	23.8	41	29.5
47–53	62	23.4	31	24.6	31	22.3
53–64	67	25.3	26	20.6	41	29.5
>64	65	24.5	39	31.0	26	18.7
						
*Menopausal status*						
Pre	111	41.9	51	40.5	60	43.2
Post	154	58.1	75	59.5	79	56.8
						
*Surgical tumour size*						
pT1	114	43.0	70	55.6	44	31.7
⩾pT2	139	52.5	52	41.3	87	62.6
ND	12	4.5	4	3.2	8	5.8
						
*Histological type*						
Lobular	39	14.7	18	14.3	21	15.1
Ductal	216	81.5	99	78.6	117	84.2
Others	10	3.8	9	7.1	1	0.7
						
*Lymph node status*						
0	126	47.5	126	100.0		
1–3	83	31.3			83	59.7
>3	56	21.1			56	40.3
						
*SBR grade in ductal carcinoma*						
1	31	14.4	17	17.2	14	12.0
2	114	52.8	58	58.6	56	47.9
3	57	26.4	22	22.2	35	29.9
ND	14	6.5	2	2.0	12	10.3
						
*ER status*						
Positive	222	83.8	108	85.7	114	82.0
Negative	43	16.2	18	14.3	25	18.0
						
*PgR status*						
Positive	208	78.5	106	84.1	102	73.4
Negative	57	21.5	20	15.9	37	26.6
						
*Adjuvant systemic therapy*						
None	25	9.4	25	19.8	0	
Hormone therapy	90	34.0	76	60.3	14	10.1
Chemotherapy	33	12.5	10	7.9	23	16.5
Hormone and chemotherapy	117	44.2	15	11.9	102	73.4

**Table 2:** *gabarapl1* expression in relation to the usual prognostic factors

	**All patients**			**pN0 patients**			**pN+ patients**		
	***N*=265**			***N*=126**			***N*=139**		
**Characteristics**	***n***	**Median**	***P-*value**	***n***	**Median**	***P-*value**	***n***	**Median**	***P-*value**
*Surgical tumour size*									
pT1	114	5.41		70	5.85		44	4.90	
⩾pT2	139	3.90		52	4.68		87	3.55	
ND	12		<0.001	4		0.212	8		0.013
									
*Histological type*									
Ductal	216	4.34		99	5.23		117	3.90	
Lobular	39	5.34		18	6.27		21	4.71	
Others	10		0.076	9		0.218	1		0.231
									
*Histological grade* a									
1	31	5.52		17	5.52		14	5.48	
2	114	4.96		58	6.12		56	4.33	
3	57	2.96		22	2.95		35	3.09	
ND	14		<0.001	2		<0.001	12		<0.001
									
*Node status*									
pN0	126	5.55							
pN+	139	4.01	<0.001						
									
*ER and PgR status*									
ER and PgR positive	191	5.08		97	6.07		94	4.50	
ER and/or PgR negative	74	3.24	<0.001	29	3.28	0.002	45	3.21	0.002

a Histological grade defined only in ductal carcinoma.

*P*-values correspond to Mann–Whitney test or Kruskall–Wallis test (histological grade).

**Table 3:** Cox univariate analysis for metastasis-free survival

	**All patients (*n*=265)**			**pN0 patients (*n*=126)**			**pN+ patients (*n*=139)**		
**Characteristics**	**HR**	**CI**	***P-*value**	**HR**	**CI**	***P-*value**	**HR**	**CI**	***P-*value**
*Age (years)*									
<48	3.22	1.50–6.93		6.29	0.73–54.16		2.42	1.06–5.54	
48–53	1.30	0.55–3.09		2.18	0.20–24.09		1.06	0.42–2.69	
54–64	0.67	0.26–1.69		1.76	0.16–19.64		0.42	0.15–1.15	
>64	1.00		<0.001	1.00		0.202	1.00		<0.001
									
*Menopausal status*									
Pre	1.65	0.97–2.82		3.60	0.93–13.97		1.40	0.77–2.52	
Post	1.00		0.066	1.00		0.064	1.00		0.271
									
*Surgical tumour size*									
pT1	1.00			1.00			1.00		
⩾pT2	3.64	1.83–7.25	0.001	1.80	0.50–6.44	0.365	3.57	1.50–8.45	0.004
									
*Histological type*									
Lobular	1.00			1.00			1.00		
Ductal	0.95	0.47–1.95	0.895	1.99	0.25–15.76	0.516	0.81	0.38–1.75	0.592
									
*Lymph node status*									
pN0	1.00								
pN+	3.67	1.85–7.30	<0.001						
									
*Histological grade* a									
1	1.00			1.00					
2	6.06	0.82–44.93		0.80	0.09–7.21				
3	10.90	1.46–81.46	0.004	1.71	0.19–15.44	0.494			NA^b^
									
*ER and PgR status*									
ER and PgR positive	1.00		0.001	1.00		0.261	1.00		<0.001
ER and/or PgR negative	2.47	1.44–4.23		0.31	0.04–2.42		3.43	1.89–6.23	
									
*Gabarapl1* status									
Log (1/*gabarapl1*)^c^	4.96	2.43–10.12	<0.001	1.32	0.20–8.92	0.777	14.96	4.80–46.60	<0.001

Abbreviations: HR=hazard ratio; CI=confidence interval; ER= oestrogen receptor; PgR=progesterone receptor.

*P-*values correspond to Cox regression model.

a Histological grade defined only in ductal carcinoma.

b No events in histological grade 1 tumour subset.

c HR for an increase of one log (1/*gabarapl1*).

**Table 4:** Cox multivariate analysis of metastasis-free survival

	**All patients (*n*=253)**			**pN0 patients (*n*=122)**			**pN+ patients (*n*=131)**		
**Characteristics**	**HR**	**CI**	***P-*value**	**HR**	**CI**	***P-*value**	**HR**	**CI**	***P-*value**
*Basal model*									
Surgical tumour size									
pT1	1.00			1.00			1.00		
⩾pT2	3.00	1.49–6.00	0.002	1.81	0.51–6.48	0.360	3.59	1.51–8.54	0.004
Lymph node status									
pN0	1.00								
pN+	2.93	1.46–5.88	0.002						
ER and PgR status									
ER and PgR positive	1.00			1.00			1.00		
ER and/or PgR negative	2.15	1.24–3.75	0.007	0.35	0.04–2.80	0.325	2.92	1.56–5.46	0.001
									
*Basal model and gabarapl1*									
Surgical tumour size									
pT1	1.00			1.00			1.00		
⩾pT2	2.68	1.33–5.42	0.006	1.62	0.44–5.95	0.464	3.21	1.34–7.70	0.009
Lymph node status									
pN0	1.00								
pN+	3.12	1.54–6.309	0.002						
ER and PgR status									
ER and PgR positive	1.00			1.00			1.00		
ER and/or PgR negative	1.73	0.96–3.12	0.007	0.21	0.02–2.27	0.200	2.41	1.26–4.62	0.008
*Gabarapl1* status									
Log (1/*gabarapl1*)^a^	3.63	1.48–8.93	0.005	3.79	0.32–45.64	0.294	5.65	1.84–17.29	0.002

Abbreviations: HR=hazard ratio; CI=confidence interval; ER= oestrogen receptor; PgR=progesterone receptor.

All values were adjusted by age.

a HR for an increase of one log /*gabarapl1*).

*P*-values correspond to Cox regression model.

## References

[bib1] Aapro MS (2001) Adjuvant therapy of primary breast cancer: a review of key findings from the 7th International Conference, St Gallen, February 2001. Oncologist 6: 376–385 11524557 10.1634/theoncologist.6-4-376

[bib2] Berthier A, Girard C, Grandvuillemin A, Muyard F, Skaltsounis AL, Jouvenot M, Delage-Mourroux R (2007) Effect of 7-O-beta-D-glucopyranosylchrysin and its aglycone chrysin isolated from Podocytisus caramanicus on estrogen receptor alpha transcriptional activity. Planta Med 73: 1447–1451 17948189 10.1055/s-2007-990248

[bib3] Bloom HJ, Richardson WW (1957) Histological grading and prognosis in breast cancer; a study of 1409 cases of which 359 have been followed for 15 years. Br J Cancer 11: 359–377 13499785 10.1038/bjc.1957.43 PMC2073885

[bib4] Chen C, Li JG, Chen Y, Huang P, Wang Y, Liu-Chen LY (2006) GEC1 interacts with the kappa opioid receptor and enhances expression of the receptor. J Biol Chem 281: 7983–7993 16431922 10.1074/jbc.M509805200

[bib5] Descotes F, Riche B, Saez S, De Laroche G, Datchary J, Roy P, André J, Bobin JY (2008) Plasminogen activator inhibitor type 1 is the most significant of the usual tissue prognostic factors in node-negative breast ductal adenocarcinoma independent of urokinase-type plasminogen activator. Clin Breast Cancer 8: 168–177 18621614 10.3816/CBC.2008.n.018

[bib6] EORTC (1980) Revision of the standards for the assessment of hormone receptors in human breast cancer; report of the second E.O.R.T.C. Workshop, held on 16–17 March, 1979, in the Netherlands Cancer Institute. Eur J Cancer 16: 1513–1515 6262087 10.1016/0014-2964(80)90064-x

[bib7] Klebig C, Seitz S, Arnold W, Deutschmann N, Pacyna-Gengelbach M, Scherneck S, Petersen I (2005) Characterization of {gamma}-aminobutyric acid type A receptor-associated protein, a novel tumor suppressor, showing reduced expression in breast cancer. Cancer Res 65: 394–400 15695379

[bib8] Lo PK, Sukumar S (2008) Epigenomics and breast cancer. Pharmacogenomics 9: 1879–1902 19072646 10.2217/14622416.9.12.1879 PMC2633440

[bib9] Mansuy V, Boireau W, Fraichard A, Schlick JL, Jouvenot M, Delage-Mourroux R (2004) GEC1, a protein related to GABARAP, interacts with tubulin and GABA(A) receptor. Biochem Biophys Res Commun 325: 639–648 15530441 10.1016/j.bbrc.2004.10.072

[bib10] Morel C, Adami P, Musard JF, Duval D, Radom J, Jouvenot M (2007) Involvement of sulfhydryl oxidase QSOX1 in the protection of cells against oxidative stress-induced apoptosis. Exp Cell Res 313: 3971–3982 17927979 10.1016/j.yexcr.2007.09.003

[bib11] Nemos C, Mansuy V, Vernier-Magnin S, Fraichard A, Jouvenot M, Delage-Mourroux R (2003) Expression of gec1/GABARAPL1 *vs* GABARAP mRNAs in human: predominance of gec1/GABARAPL1 in the central nervous system. Brain Res Mol Brain Res 119: 216–219 14625090 10.1016/j.molbrainres.2003.09.011

[bib12] Pellerin I, Vuillermoz C, Jouvenot M, Ordener C, Royez M, Adessi GL (1993) Identification and characterization of an early estrogen-regulated RNA in cultured guinea-pig endometrial cells. Mol Cell Endocrinol 90: R17–R21 8495796 10.1016/0303-7207(93)90161-c

[bib13] Roberts SS, Mori M, Pattee P, Lapidus J, Mathews R, O’Malley JP, Hsieh YC, Turner MA, Wang Z, Tian Q, Rodland MJ, Reynolds CP, Seeger RC, Nagalla SR (2004) GABAergic system gene expression predicts clinical outcome in patients with neuroblastoma. J Clin Oncol 22: 4127–4134 15483022 10.1200/JCO.2004.02.032

[bib14] Sasco AJ, Kaaks R, Little RE (2003) Breast cancer: occurrence, risk factors and hormone metabolism. Expert Rev Anticancer Ther 3: 546–562 12934666 10.1586/14737140.3.4.546

[bib15] Sobin LH, Wittekind C (eds) (1997) Breast tumours (ICD-O C50). In TNM Classification of Malignant Tumours, 5 edn, pp 123–130. Wiley-Liss: New York

[bib16] Tolle F, Risold PY, Mansuy-Schlick V, Rossi E, Boyer-Guittaut M, Fraichard A, Jouvenot M (2008) Specific regional distribution of gec1 mRNAs in adult rat central nervous system. Brain Res 1210: 103–115 18423580 10.1016/j.brainres.2008.02.077

[bib17] Tomlinson IP (2001) Mutations in normal breast tissue and breast tumours. Breast Cancer Res 3: 299–303 11597318 10.1186/bcr311 PMC138692

[bib18] Towbin H, Staehelin T, Gordon J (1979) Electrophoretic transfer of proteins from polyacrylamide gels to nitrocellulose sheets: procedure and some applications. Proc Natl Acad Sci USA 76: 4350–4354 388439 10.1073/pnas.76.9.4350 PMC411572

